# An investigation of the conformity, feasibility, and expected clinical benefits of multiparametric MRI-guided dose painting radiotherapy in glioblastoma

**DOI:** 10.1093/noajnl/vdac134

**Published:** 2022-08-19

**Authors:** Caterina Brighi, Paul J Keall, Lois C Holloway, Amy Walker, Brendan Whelan, Philip C de Witt Hamer, Niels Verburg, Farhannah Aly, Cathy Chen, Eng-Siew Koh, David E J Waddington

**Affiliations:** ACRF Image X Institute, Sydney School of Health Sciences, Faculty of Medicine and Health, The University of Sydney, Sydney, Australia; Ingham Institute for Applied Medical Research, Sydney, Australia; ACRF Image X Institute, Sydney School of Health Sciences, Faculty of Medicine and Health, The University of Sydney, Sydney, Australia; Ingham Institute for Applied Medical Research, Sydney, Australia; Ingham Institute for Applied Medical Research, Sydney, Australia; Department of Radiation Oncology, Liverpool and Macarthur Cancer Therapy Centres, Liverpool, Australia; Centre for Medical Radiation Physics, University of Wollongong, Wollongong, Australia; Ingham Institute for Applied Medical Research, Sydney, Australia; Department of Radiation Oncology, Liverpool and Macarthur Cancer Therapy Centres, Liverpool, Australia; Centre for Medical Radiation Physics, University of Wollongong, Wollongong, Australia; ACRF Image X Institute, Sydney School of Health Sciences, Faculty of Medicine and Health, The University of Sydney, Sydney, Australia; Ingham Institute for Applied Medical Research, Sydney, Australia; Brain Tumor Center Amsterdam, Amsterdam UMC, Amsterdam, The Netherlands; Department of Neurosurgery, Amsterdam UMC, Amsterdam, The Netherlands; Brain Tumor Center Amsterdam, Amsterdam UMC, Amsterdam, The Netherlands; Department of Neurosurgery, Amsterdam UMC, Amsterdam, The Netherlands; Ingham Institute for Applied Medical Research, Sydney, Australia; Department of Radiation Oncology, Liverpool and Macarthur Cancer Therapy Centres, Liverpool, Australia; Department of Radiation Oncology, Liverpool and Macarthur Cancer Therapy Centres, Liverpool, Australia; Ingham Institute for Applied Medical Research, Sydney, Australia; Department of Radiation Oncology, Liverpool and Macarthur Cancer Therapy Centres, Liverpool, Australia; ACRF Image X Institute, Sydney School of Health Sciences, Faculty of Medicine and Health, The University of Sydney, Sydney, Australia; Ingham Institute for Applied Medical Research, Sydney, Australia

**Keywords:** dose painting, glioblastoma, MRI-guided radiotherapy, multiparametric MRI, planning study

## Abstract

**Background:**

New technologies developed to improve survival outcomes for glioblastoma (GBM) continue to have limited success. Recently, image-guided dose painting (DP) radiotherapy has emerged as a promising strategy to increase local control rates. In this study, we evaluate the practical application of a multiparametric MRI model of glioma infiltration for DP radiotherapy in GBM by measuring its conformity, feasibility, and expected clinical benefits against standard of care treatment.

**Methods:**

Maps of tumor probability were generated from perfusion/diffusion MRI data from 17 GBM patients via a previously developed model of GBM infiltration. Prescriptions for DP were linearly derived from tumor probability maps and used to develop dose optimized treatment plans. Conformity of DP plans to dose prescriptions was measured via a quality factor. Feasibility of DP plans was evaluated by dose metrics to target volumes and critical brain structures. Expected clinical benefit of DP plans was assessed by tumor control probability. The DP plans were compared to standard radiotherapy plans.

**Results:**

The conformity of the DP plans was >90%. Compared to the standard plans, DP (1) did not affect dose delivered to organs at risk; (2) increased mean and maximum dose and improved minimum dose coverage for the target volumes; (3) reduced minimum dose within the radiotherapy treatment margins; (4) improved local tumor control probability within the target volumes for all patients.

**Conclusions:**

A multiparametric MRI model of GBM infiltration can enable conformal, feasible, and potentially beneficial dose painting radiotherapy plans.

**Key Points**

- Multiparametric MRI-guided dose painting radiotherapy is feasible in glioblastoma.
- Dose painting could improve local control without increasing normal tissue dose.
- A clinical treatment planning system can generate conformal dose painting plans.

**Importance of the Study**

Glioblastoma infiltration and heterogeneous radiosensitivity limit local control rates with current standard of care treatment. Multiparametric MRI enables quantification of physiological processes linked to tumor infiltration and radiosensitivity, providing opportunities for personalized dose painting radiotherapy and the potential to improve local control and survival outcomes. Practical integration of functional imaging information into optimal radiotherapy plans at the voxel level remains a challenge for clinical implementation. We demonstrate an end-to-end clinical workflow for the generation of dose painting radiotherapy plans from a per-voxel multiparametric MRI model of glioma infiltration. We show that dose painting is feasible and has likely clinical benefits. We discuss future steps required to help design future dose painting phase II clinical trials. These steps include validation studies evaluating the relevance of the model to target likely areas of tumor recurrence and observational studies identifying imaging biomarkers for patient stratification and adaptive treatment strategies evaluation.

Glioblastoma multiforme (GBM) is one the most aggressive types of brain cancer, for which treatment efficacy remains extremely limited. Only 5.1% of GBM patients survive 5-years post diagnosis, and 63% develop tumor recurrence within 1-year following standard of care treatment, which involves maximal safe surgical resection followed by fractionated radiotherapy (60 Gy delivered in 30 daily fractions) with concurrent and adjuvant chemotherapy.^1^ Progress in the treatment of GBM has stalled since 2005 when it was found that radiotherapy delivered concomitantly with temozolomide (TMZ) chemotherapy leads to significant survival benefits over radiotherapy alone.^2^ Over the past two decades, numerous studies have worked to improve treatment outcomes via different approaches, including the development of new targeting chemotherapeutic agents, cutting-edge surgical techniques, and radiotherapy dose escalation approaches. None of these approaches has resulted in significant survival improvements, reflecting the severity of the challenges posed by the complexity of this disease.^3,4^

Tumor progression remains the major cause of treatment failure, with >70–80% of tumors recurring locally at the site of the primary lesion.^5,6^ Tumor progression is caused by the intrinsic heterogeneity of GBM at the molecular, cellular, and tissue level, which enables GBM cells to develop mechanisms of treatment resistance.^7^ Radio-resistance, which is the ability of certain subpopulations of GBM cells to withstand ionizing radiation, is linked to the development of local recurrence even after radiation treatment with dose sufficient to kill the bulk of tumor cells. Cell death in radio-resistant cells can require up to three times the dose of radiation compared to radio-sensitive cells.^8^

This knowledge has motivated a wave of prospective clinical trials aimed at evaluating dose escalation strategies to improve local control and, ultimately, clinical outcomes. The results of these studies, which have been discussed in a recent systematic review and meta-analysis by Singh et al.,^9^ revealed that dose escalation provides survival benefits over conventional radiation treatment only when used alone (ie without concomitant TMZ) and to subpopulations of patients with an unmethylated DNA repair enzyme O[6]-methylguanine-DNA methyltransferase.

Most of the dose escalation studies analyzed in the review involved the delivery of an equivalent dose up to 81.6 Gy via hypo-fractionated intensity modulated radiation therapy with a simultaneous integrated boost technique to high-risk areas. The high-risk area was most commonly defined as the postoperative cavity and residual disease identified on a T_1_-weighted contrast enhanced (T1CE) MRI sequence with the addition of a 0.5–1 cm margin.^9^ The selection of this high-risk area as a target for dose escalation has been driven by historical observations of recurrence developing most often in this region. However, this approach to target definition for dose escalation neglects the role of biological heterogeneity characterizing the tumor microenvironment, which is known to play a key role in treatment resistance leading to local recurrence.^7,10^

Biological heterogeneity, which encompasses different aspects of the physiology of the tumor microenvironment – including vascular perfusion, cellularity, oxygenation status, and metabolism – is poorly captured by imaging techniques currently used for treatment planning and response assessment. Standard imaging techniques include T1CE and fluid-attenuated inversion recovery (FLAIR) MRI, which only provide anatomical information of the size and shape of the bulk of the tumor and an approximate estimate of its volumetric impact on the surrounding brain tissue. Characterization of biological heterogeneity requires more sophisticated MRI or amino acid positron emission tomography (PET) imaging techniques.^11,12^ Using these more advanced functional imaging techniques to redefine the dose distribution within the radiotherapy target volume could bring previously unachieved clinical benefits stemming from improved local control, by selectively targeting regions of the tumor that are responsible for evolving treatment resistance with higher doses of radiation, while sparing normal brain from unnecessary toxicity.^13–15^ This approach, which involves the prescription and delivery of a nonuniform radiation dose distribution to the target volume based on physiological information of the tumor microenvironment, is known as dose painting (DP) (Figure 1).^16^ Identifying the imaging modality, or the combination thereof, that best correlates to regions of treatment resistance is paramount to the success of DP radiotherapy.

**Figure 1.**
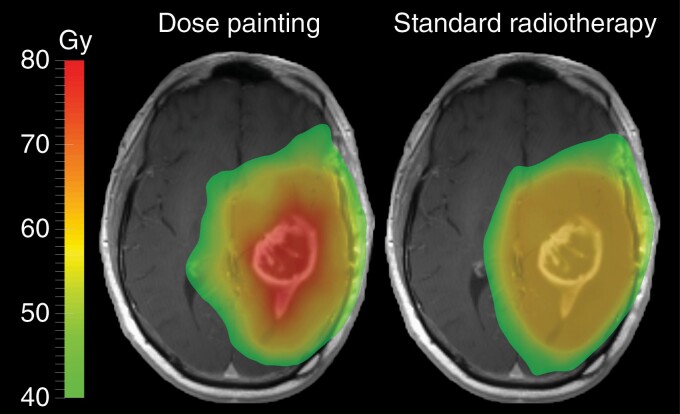
Example of dose painting versus standard radiotherapy. Dose painting involves modulating the dose based on physiological information of the tumor microenvironment. Standard radiotherapy involves delivering a uniform dose of 60 Gy to the radiotherapy target.

Kim et al.^17^ recently demonstrated the relevance of diffusion- and perfusion-weighted MRI in the identification of relevant regions of treatment resistance that evolve during radiation treatment correlating with worse survival outcomes. These regions extend outside the conventionally defined dose escalation target volume and could benefit from dose escalation. Importantly, in a phase II trial they showed that escalating the dose to 76 Gy in these regions improved 1-year overall survival rate by 27% compared to standard treatment outcomes from historical controls.^18^ As such, the combination of these two advanced MRI techniques could represent the best approach for DP adaptive planning strategies that aim to adjust the radiotherapy plan over the course of treatment in response to the evolving physiology in the tumor microenvironment.

To integrate the use of these two imaging modalities into routine radiotherapy planning, clinically feasible workflows must be developed. In this study we present an end-to-end clinical workflow (Figure 2) to integrate a multiparametric MRI model of tumor infiltration into the development of DP plans in GBM patients. This model, which combines diffusion- and perfusion-weighted MRI data to generate per-voxel predictions of tumor infiltration probability, was previously biologically validated with histopathology from preoperative image-guided stereotactic biopsy as reference standard,^19^ and was proven to yield reliable DP prescriptions.^20^ Overall, we aim to demonstrate the feasibility of delivering DP plans in a clinical treatment planning system and the potential of this approach to improve local control in GBM patients.

**Figure 2.**
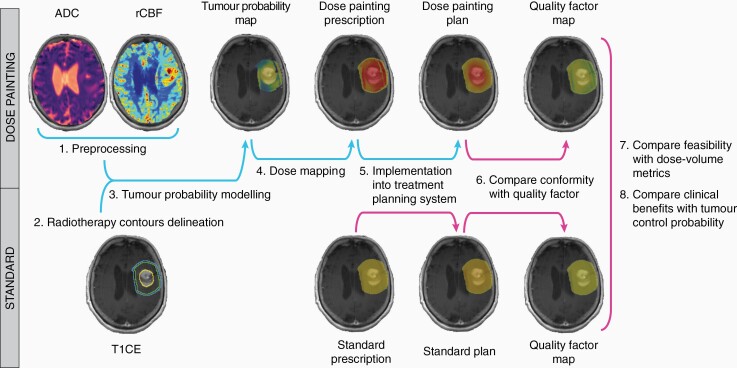
Diagram of clinical workflow for dose painting and comparative analyses with standard radiotherapy. Clinical workflow: steps 1–5. Comparative analyses: steps 6–8. 1. Preprocessing diffusion-/perfusion-weighted MRI data. 2. Radiotherapy contours delineation from T1CE. 3. Tumor probability modeling within the radiotherapy target volume. 4. Per-voxel conversion of tumor probability into dose prescription. 5. Import of dose prescriptions into treatment planning system and generation of dose painting plans. 6. Assessment of plans conformity to dose prescriptions via quality factor. 7. Assessment of feasibility of dose painting plans via dose-volume metrics to target volumes and organs at risk. 8. Assessment of potential clinical benefits by comparison of tumor control probability between dose painting and standard radiotherapy plans. ADC, apparent diffusion coefficient; rCBF, relative cerebral blood flow; T1CE, T_1_-weighted contrast enhanced image.

## Materials and Methods

### Patient Dataset

Anonymized, publicly available data from the QIN GBM Treatment Response collection on The Cancer Imaging Archive (originally submitted under IRB-approved protocol) were used for this study.^21–24^ The dataset consists of MR images of 54 newly diagnosed GBM patients acquired 3–7 days prior to commencement of postoperative (3–5 weeks post-surgery) chemoradiation treatment.^25^ Images used in this study included T1CE, FLAIR, apparent diffusion coefficient (ADC) maps derived from diffusion-weighted MRI, and relative cerebral blood flow (rCBF) maps derived from dynamic-susceptibility contrast (DSC) MRI. An inclusion criterion for this study was the availability of both an ADC map and DSC MRI sequence in the patient’s dataset, and only 17 of 54 patients fit this criterion. Imaging acquisition parameters can be found on the data collection webpage^21^ and in Supplementary Table S1.

### Image Preprocessing

**Figure 3.**
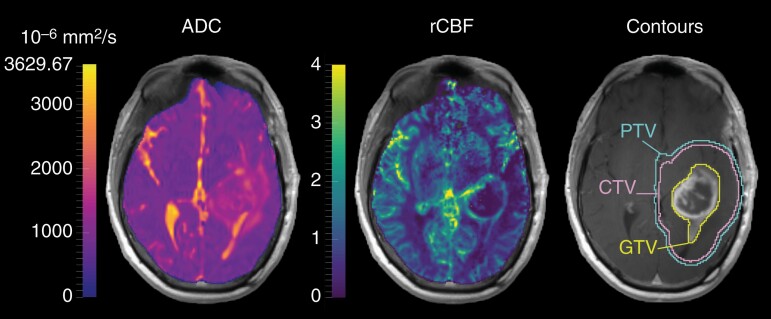
Parametric maps and radiotherapy contours. Example of T_1_-weighted image with overlayed apparent diffusion coefficient (ADC) map, relative cerebral blood flow (rCBF) map, and radiotherapy contours. CTV, clinical target volume; GTV, gross tumor volume; PTV, planning tumor volume.

NordicICE (v4.1.3, NordicNeuroLab) was used to rigidly register FLAIR, ADC, and DSC images to the T1CE, and to derive a rCBF map from the DSC sequence following consensus recommendations for DSC MRI analysis in high-grade gliomas^24,26^ Binary brain masks were generated from the T1CE, visually inspected and manually adjusted.^27,28^ All images were resampled to 1.2 mm isotropic resolution.^29^ A mask of the brain volume covered by the field of view in the DSC sequence was generated and used to derive parametric maps only in regions of the brain where both diffusion- and perfusion-weighted data were acquired (Figure 3). The resulting ADC and rCBF maps were normalized to a reference volume manually selected in the contralateral normal brain, smoothed with an edge-preserving bilateral filter, and standardized, as per the method in Verburg et al.^19^ Binary masks of the cerebrospinal fluid (CSF) volume were derived from FLAIR images.^30^

### Radiotherapy Contours Delineation

Radiotherapy contours delineation was performed by a radiation oncologist using the software MIM following EORTC guidelines^31,32^ and contours were reviewed by a second senior radiation oncologist. The gross tumor volume (GTV) was defined from the T1CE and included the resection cavity and any contrast-enhancing margins. The clinical target volume (CTV) was determined by 1.5 cm expansion of the GTV and modified to anatomical boundaries, and planning target volume (PTV) by 0.3 cm expansion of the CTV (Figure 3). T1CE images with associated radiotherapy structures were imported into RayStation treatment planning system (v.10B, RaySearch Laboratories). Organs at risk (OARs) were delineated in RayStation from the T1CE image by a radiation therapist with the aid of a MRI-based OARs atlas, following EORTC guidelines.^31,32^

### Derivation of Tumor Probability Map from Diffusion and Perfusion Parameters

Intensity values from ADC and rCBF maps were linearly combined according to a biologically validated model of glioma infiltration to generate a map of per-voxel tumor probability.^19^ The coefficients used for the linear combination of ADC and rCBF values were determined with a logistic regression analysis, as it has been reported elsewhere.^20^ Briefly, the probability of tumor infiltration at each voxel, *i*, was obtained with the following formula:


$$\begin{array}{l} Tumour\;\;\;probability_{i}=1.64+1.80\cdot ADC_{i}+1.43\cdot rCBF_{i} \end{array}$$

The tumor probability maps were masked within the CTV. A tumor probability of 1 was assigned to all voxels within the GTV, which is, by definition, residual tumor. All voxels in the CSF were assigned a tumor probability of 0, as the model was not validated in this region.

### Derivation of Dose Painting Prescriptions from Tumor Probability Map

A per-voxel dose prescription within the CTV was derived from the per-voxel tumor probability by means of a linear dose mapping function, as previously described.^33^ The use of this function assumes a linear relationship between the probability of tumor presence and the rate of conferred radio-resistance of cells in each voxel. This assumption is often made to simplify the lack of knowledge on the exact relationship between image-derived biological information and tumor-specific radiobiological parameters of treatment response, the establishment of which remains an open challenge in this field of research.^33^ The dose prescription at each voxel, *i*, was obtained according to the following equation:


$$\begin{array}{l} Dose\;prescription_{i}=\;D_{min}+\left(D_{max}-D_{min}\right)\cdot Tumour\;probability_{i} \end{array}$$

where $D_{min}$ is the minimum prescribed dose set to 60 Gy, reflecting the standard of care adjuvant radiation dose recommended by EORTC guidelines for young, fit patients with GBM.^32^$D_{max}$ is the maximum dose set to 80 Gy, corresponding to the maximum tolerated dose considered safe in the RTOG dose escalation trial 98-03.^34^ All voxels within the margins of expansion between the CTV to the PTV were prescribed a dose of 60 Gy. To deliver a heterogeneous dose distribution to the target volume, an inverse dose optimization process previously described by Arnesen et al.^35^ was adopted. This approach, chosen for its compatibility with commercial treatment planning systems, involves the conversion of the dose prescription within the PTV into an inverse dose prescription, $D_{inv,i}$ using the following equation:


$$\begin{array}{l} D_{inv,i}=\;D_{max}-Dose\;prescription_{i} \end{array}$$

The inverse dose prescription masked to the PTV was finally imported into RayStation. The code used for image preprocessing and derivation of tumor probability maps and DP prescriptions is publicly available on our GitHub repository https://github.com/cbri92/mpMRI_TP_DP.

### Radiotherapy Treatment Planning

A standard plan and a DP plan were developed for each patient in RayStation. To overcome the absence of computed tomography (CT) data in the public dataset, water-equivalent density overrides were assigned to the brain (1.04 g/cm^3^) and the skull (1.61 g/cm^3^). Both plans were created as volumetric modulated arc therapy with two 6 MV full arcs from an Elekta Versa HD with Agility MLC (5 mm leaf size). For the standard plan, an isodose of 60 Gy delivered in 30 daily fractions was assigned to the PTV. Dose constraints to OARs were set according to EORTC guidelines.^31,32^ For the DP plan the inverse dose prescription map previously imported into RayStation was assigned to a mock treatment plan. DP plans were generated using an approach previously described by Arnesen et al.^35^ The optimization was performed in dose summation mode, where the mock plan containing the inverse DP prescription was assigned as a pretreated plan. As optimization targets minimum dose of 76.8 Gy to GTV, minimum dose of 61.2 Gy to CTV, minimum dose of 60.6 Gy to PTV, and maximum dose of 77.5 Gy to PTV were used to allow the optimizer to create the prescribed DP dose distribution within the PTV for the actual plan (Supplementary Fig. S1). Fractionation and dose constraints to OARs were set as per the standard plans.

### Evaluation Metrics

The conformity of the DP plan was measured with a quality factor (QF), defined as in previous studies^35–38^ as:


$$\begin{array}{l} QF=100-\frac{1}{n}\underset{i}{\sum }\;\left|\frac{Dose_{plan,i}-Dose_{prescribed,\;i}}{Dose_{prescribed,\;i}}\right|. \end{array}$$

For an ideal plan QF would equal 100%. For the DP plans, the treatment planning goal was set to QF ≥ 95% within the GTV, as proposed by Duprez et al.^38^ for DP in head and neck cancer. The metrics used to evaluate feasibility of the radiotherapy plans included dose-volume metrics to PTV, CTV, GTV, and OARs. Potential clinical benefits of the plans were assessed by means of tumor control probability (TCP) in the CTV and GTV. A computer program previously developed by our group was used to calculate dose-volume metrics to the radiotherapy target volumes and OARs from dose-volume histogram files.^39^ The dose metrics evaluated included: dose to 98% volume, D_98% vol_, mean dose, D_mean_, maximum dose, D_max_, volume receiving 60 Gy, V_60Gy_, volume receiving 76 Gy, V_76Gy_, in PTV, minimum dose, D_min_, in GTV, D_mean_ in the brain, and D_max_ in the brainstem, chiasm, lenses, optic nerves, and retinas. To calculate the TCP, a previously developed model converting the dose distribution into a TCP was used.^40^ In this model TCP within a target volume is defined by the following equation:


$$\begin{array}{l} TCP=e^{-C\sum_{i}p_{i}e^{-\alpha D_{i}}} \end{array}$$

where $p_{i}$ is the probability of tumor presence and $D_{i}$ is the dose prescription at voxel *i*, α is the intrinsic tumor radiosensitivity and C is a constant. α = 0.12 Gy^−^1 was selected based on an estimate from historical dose escalation clinical studies in GBM,^41^ and C = 0.032 was estimated by fitting the standard radiotherapy plans data such that the average TCP in the CTV over the entire patient population reflected the 1-year progression free survival rate for standard of care treatment reported in the Stupp study (ie 27%).^2^

### Statistical Analysis

Statistical analysis was performed using GraphPad Prism (v9.1.2). A pairwise comparison between the standard and the DP plans was performed via a Wilcoxon matched-pairs signed-rank test for each evaluation metric.

## Results

### Radiotherapy Planning Workflow and Quality Assessment of the Plans

**Figure 4.**
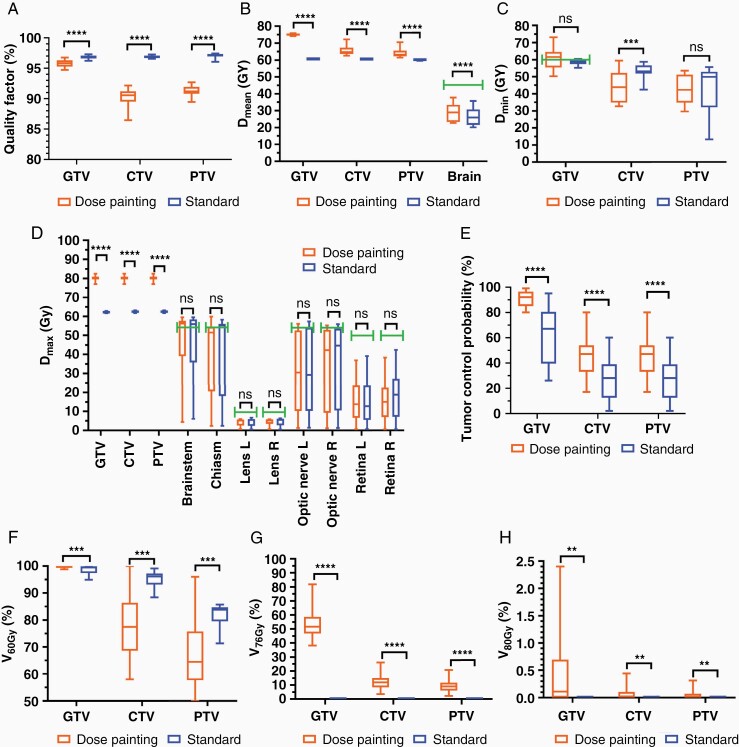
Quantitative analysis of plans evaluation metrics and comparison between standard and dose painting plans. A. Quality factor, B. mean dose (*D*_mean_), C. minimum dose (*D*_min_), and D. maximum dose (*D*_max_) within radiotherapy targets and organs at risk. E. Tumor control probability (TCP) within GTV and CTV, F. percentage volume receiving 60 Gy (*V*_60Gy_), G. percentage volume receiving 76 Gy (*V*_76Gy_), and H. percentage volume receiving 80 Gy (*V*_80Gy_) within radiotherapy targets. GTV, gross tumor volume; CTV, clinical target volume; PTV, planned target volume. Bar charts show mean and standard deviation. Bars represent dose constraints to organs at risk: *D*_mean_ < 45 Gy for brain, *D*_max_ < 54 Gy for brainstem, chiasm, and optic nerves, *D*_max_ < 10 Gy for lenses, and *D*_max_ < 50 Gy for retina. *P < .05, **P < .01, ***P < .001, ****P < .0001, ns = no significant difference. *N* = 17.

**Figure 5.**
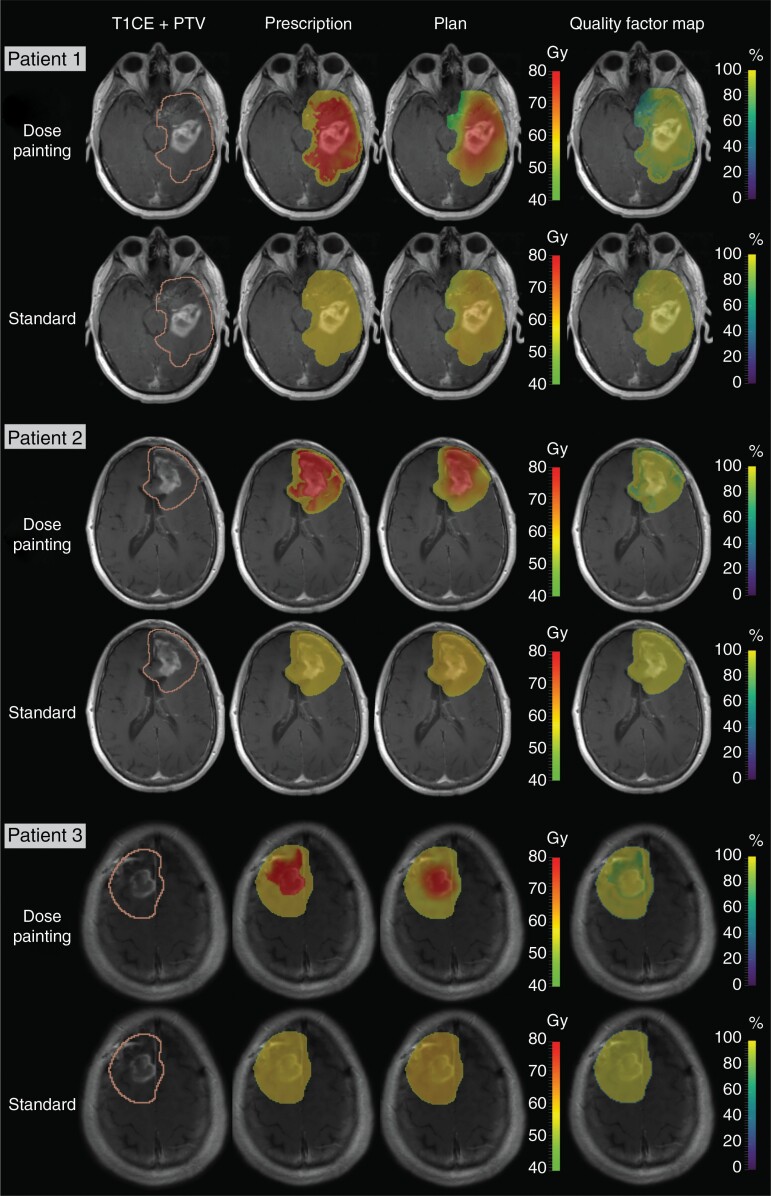
Examples of comparison between standard and dose painting plans. The figure shows T1CE with overlayed PTV, dose prescriptions, dose plans, and quality factor maps in the PTV for the standard and the dose painting plans for 3 patients. T1CE, T_1_-weighted contrast enhanced image; PTV, planning target volume.

DP plans were successfully generated from the DP prescriptions using the clinical workflow presented in this study (Figure 2). Both DP and standard plans had high conformity, with mean QF of 95.8 ± 0.6%, 90.3 ± 1.3%, and 91.2 ± 0.8% (DP plans) and 96.8 ± 0.3%, 96.9 ± 0.2%, and 97.0 ± 0.4% (standard plans) within the GTV, CTV, and PTV, respectively (Figure 4). The conformity of the standard plans was significantly higher than for the DP plans (*P* < .0001; Figure 4A). Figure 5 shows dose prescriptions, plans, and QF maps for three patients randomly selected from the study cohort. The conformity of the DP plans was lower for voxels extending from the GTV to the CTV margins compared to voxels within the GTV.

### Quantitative Comparison between Standard and Dose Painting Plans

DP did not significantly increase D_max_ delivered to the OARs, showing lower or equal values compared to the respective standard plans (*P* > .05; Figure 4D). It is worth noting that when dose constraints were exceeded, this occurred for both the standard and the DP plans. For both plans, D_max_ mostly stayed within the dose constraints for OARs, with 10 patients receiving up to 5 Gy higher dose than 54 Gy to the brainstem and chiasm, and 4 patients (standard) and 2 patients (DP) receiving up to 3 Gy higher dose than 54 Gy to the optic nerves. While D_mean_ to the brain was significantly increased in the DP plans compared to the respective standard plans (*P* < .0001; Figure 4B), these values were still significantly lower than the dose constraint of D_mean_ < 50 Gy (*P* < .0001; Figure 4B).

DP significantly increased D_mean_ and D_max_ within all radiotherapy targets (*P* < .0001; Figure 4B and D), with mean values of D_mean_ = 75.1 ± 0.5 Gy and D_max_ = 80.1 ± 1.2 Gy within the GTV across patients, and significantly increased TCP by 18% within the GTV and by 29% within the CTV (*P* < .0001; Figure 4E) compared to standard radiotherapy. Additionally, DP resulted in higher values of D_min_ to the GTV (60.6 ± 6.0 Gy vs 58.6 ± 1.7 Gy, *P* = .243; Figure 4C) and lower values of D_min_ within the CTV and PTV (*P* = .001 and *P* = .579, respectively; Figure 4C) compared to the standard plans. Similarly, DP resulted in significantly higher V_60Gy_ within the GTV (99.9 ± 0.3% vs 98.7 ± 1.6%, *P* = .0002; Figure 4F), and significantly lower V_60Gy_ within the CTV and PTV (*P* = .0003 and *P* = .0008, respectively; Figure 4F) compared to the respective standard plans. Furthermore, the standard approach resulted in zero values of V_76Gy_ and V_80Gy_ within all radiotherapy targets for all patients, while DP significantly increased values of V_76Gy_ and V_80Gy_ within all target volumes (*P* < .0001 and *P* < .01, respectively; Figure 4E and H). With DP, V_76Gy_ reached mean values of 53.7 ± 10.2%, 12.3 ± 5.5%, and 9.3 ± 4.4%, and V_80Gy_ reached mean values of 0.4 ± 0.7%, 0.07 ± 0.12% and 0.05 ± 0.09% within the GTV, CTV, and PTV, respectively.

## Discussion

Integrating voxel-level physiological information from functional imaging into radiotherapy plans optimization has long been a practical challenge for DP. In this study we presented an end-to-end clinical workflow for the generation of DP plans from a per-voxel multiparametric MRI model of tumor infiltration combining diffusion- and perfusion-weighted MRI in GBM. We showed that this approach enables the generation of DP plans with high conformity, with average QF values exceeding the set target of 95% within the GTV. This result demonstrates that using per-voxel imaging information to plan the delivery of a heterogeneous dose distribution is technically feasible on a clinical treatment planning system, and that the resulting plans are in good agreement with the prescribed dose distributions. As illustrated in Figure 5, voxels with lower QF are mostly found in the region extending from the GTV to the CTV margins, reflecting the technical limitations of the photon beam properties and the multi-leaf collimator resolution restricting the achievement of the prescribed heterogeneous dose distribution at the dose prescription map resolution (ie 1.2 mm isotropic).^42,43^ While, to our knowledge, no other studies have reported QF of DP plans in newly diagnosed GBM, other studies have reported similar QF values for MRI/PET-guided DP in recurrent GBM, cervix, head and neck, lung, and prostate cancers.^35–38^

We then demonstrated that, compared to the standard approach, DP allows an increase in D_mean_ and D_max_ to the radiotherapy target volumes, specifically targeting regions likely harboring radioresistant tumor, without increasing the dose to OARs. The values of D_min_ and V_60Gy_ achieved with DP, which were higher in the GTV and lower in the CTV and PTV compared to the standard plans, demonstrated the ability of DP to better fulfill the minimum dose coverage requirement for the GTV and reduce the dose within radiotherapy margins of uncertainty. Additionally, results on values of V_76Gy_ and V_80Gy_ further attest to the feasibility of this DP approach, which allows targeting of only a small portion of the PTV with high radiation doses. These results are particularly important for providing evidence of the feasibility of testing this approach in a phase I clinical trial, where radiation safety and toxicity would be the primary objective evaluated. Jena et al.^44^ demonstrated similar results in a planning study using diffusion-tensor imaging to guide dose escalation in GBM by means of reducing the radiotherapy target volume compared to the standard approach. Dose escalation in the range 64–74 Gy was possible without increasing normal tissue complication probability compared to the respective standard plans.

While our study does not provide clinical evidence that boosting the dose above 60 Gy based on multiparametric MRI will provide survival benefits over standard treatment by improving local tumor control, our TCP estimates suggest that this is a likely outcome. This hypothesis, which remains to be validated clinically, is supported by evidence provided by a recent phase II clinical trial.^18^ Using a combination of diffusion- and perfusion-weighted MRI-derived parameters to guide dose escalation to 76 Gy – a similar approach to the one used in our study – Kim et al.^18^ demonstrated a 27% improvement in 12-month overall survival in the dose escalation arm compared to historical controls.

The conclusions that can be drawn from this study are limited by the available data and the retrospective nature of our experiments. We currently have no evidence that the multiparametric MRI model of tumor infiltration used in this study spatially predicts areas of tumor recurrence. As this model was biologically validated on pre-operative image-guided biopsies, clinical validation of this point is necessary to provide further evidence for the assumption that selective dose escalation in regions that are predicted to have a high probability of tumor infiltration would lead to improved local tumor control. Such evidence could be provided by investigating the spatial correlation between the tumor probability maps generated from preradiotherapy imaging and the patterns of progression of recurrent tumor observed on follow-up MRI scans, as previously performed.^45^ However, it is worth noting that there are some limitations linked to this validation method, namely the challenge of distinguishing recurrent tumor from treatment-induced radiological changes on current anatomical follow-up imaging, the large variation in radiation treatment response observed between patients, brain tissue deformations due to surgery, tumor mass effects, and treatment-related brain atrophy. While addressing these limitations in their complexity is beyond the scope of this article, there is evidence suggesting that the addition of perfusion- and diffusion-weighted MRI at follow-up imaging could help differentiating between recurrent tumor and radiation-induced radiological changes.^46^

Another limitation of this study, and of other retrospective planning studies evaluating strategies for image-guided DP in GBM, is the assumption that the encouraging results suggesting improved local control rates and acceptable levels of toxicity will apply to every GBM patient. Unfortunately, this is not the case, as response to radiotherapy dose escalation varies substantially between GBM patients.^9^ Thus, research studies focusing on the validation of imaging biomarkers that help identify groups of patients likely to benefit from emerging image-guided DP radiotherapy approaches is paramount for the establishment of appropriate inclusion criteria for future phase II clinical trials. Such validation studies require longitudinal assessments of the changes in imaging biomarkers observed during radiotherapy and investigating their correlation with clinical outcomes of progression-free survival and overall survival. Imaging biomarkers derived from diffusion- and perfusion-weighted MRI have recently been shown to help identify GBM patients who would benefit from an image-guided DP approach.^17^

Another limitation of our study is that treatment effects of fractionation are not accounted for and the tumor, in its spatial heterogeneity, is considered as a static entity during treatment. However, we know that this is not the case, as GBM tumor physiology – including cellular density, vascular perfusion, and radiosensitivity – varies during the course of radiotherapy, and changes in these processes in response to radiotherapy also affect the overall treatment efficacy.^17^ As such, it is necessary to take these physiological changes into account during radiation treatment, by adopting adaptive treatment strategies. Ideally, tumor probability maps should be generated at the beginning and at several timepoints during treatment, such that to enable adaptation of DP prescriptions during fractionation according to tumor response to treatment. MRI-Linac systems, which provide imaging capabilities during a course of treatment and are rapidly being clinically deployed worldwide, offer a tangible opportunity to make biologically-adaptive MRI-guided radiotherapy the next frontier in personalized brain cancer radiatiotherapy.^47^

Finally, the additional time and cost of running diffusion- and perfusion-weighted sequences could limit the feasibility of applying this technique at clinical sites where patients have limited access to MRI systems. While this is an important consideration, most clinical centers with a radiation oncology department around the world have access to an MRI system, which is required for treatment planning. Acquiring these sequences only takes ~5–7 min over routine scans, thus representing an acceptable cost for the socio/economic benefit of extending patients’ survival time.

In conclusion, we demonstrated that a multiparametric MRI model of GBM infiltration can be used to generate conformal, feasible, and potentially beneficial DP radiotherapy plans for GBM patients. This represents a key step to the clinical implementation of this multiparametric MRI model of GBM infiltration for DP radiotherapy. Nevertheless, additional steps are needed to investigate whether this model can improve survival outcomes in GBM.

## Supplementary Material

vdac134_suppl_Supplementary_Material
