# Supplementary material for: An investigation of the conformity, feasibility, and expected clinical benefits of multiparametric MRI-guided dose painting radiotherapy in glioblastoma
Source: Neurooncol Adv. 2022 Aug 19;4(1):vdac134. doi: 10.1093/noajnl/vdac134 (PMC9466270; doi:10.1093/noajnl/vdac134)
Supplement: vdac134_suppl_Supplementary_Material [file vdac134_suppl_supplementary_material.docx]

**Supplementary Material**

**Table S1**. Imaging sequences acquisition parameters.^[[1]](#footnote-1)^

| Data | Sequence | Parameters | Notes |
| --- | --- | --- | --- |
| T1CE | 2D Fast low angle shot | TR/TE/α = 600 ms/12 ms/90°, matrix size 256 × 216, FOV 185mm × 220mm, 23 slices and 5mm slice thickness |  |
| FLAIR | 2D spin-echo inversion-recovery | TR/TI/TE = 10000 ms/2500 ms/70 ms, matrix size 256 × 162, FOV 185mm × 220mm, 23 slices and 5mm slice thickness |  |
| DWI | Echo planar imaging | Acquired with trace diffusion sensitization and b-values of 0 and 700 s/mm2, TR/TE = 7980 ms/84 ms, matrix size 128 × 128, FOV 237mm × 237mm, 64 slices and 1.86mm slice thickness | Provided data for system generated ADC maps |
| DSC | Gradient echo 2D echo planar imaging | TR/TE/α = 1500 ms/31 ms/80°, matrix size 160 × 160, FOV 192mm × 192mm, 12 slices and 5mm slice thickness. The acquisition was repeated 100 times for a total scan time of 2 min and 41 s, corresponding to a temporal resolution of 1.61 s. | A bolus of 0.1 mmol/kg of GD-DTPA was injected 80 s after the scan started. |


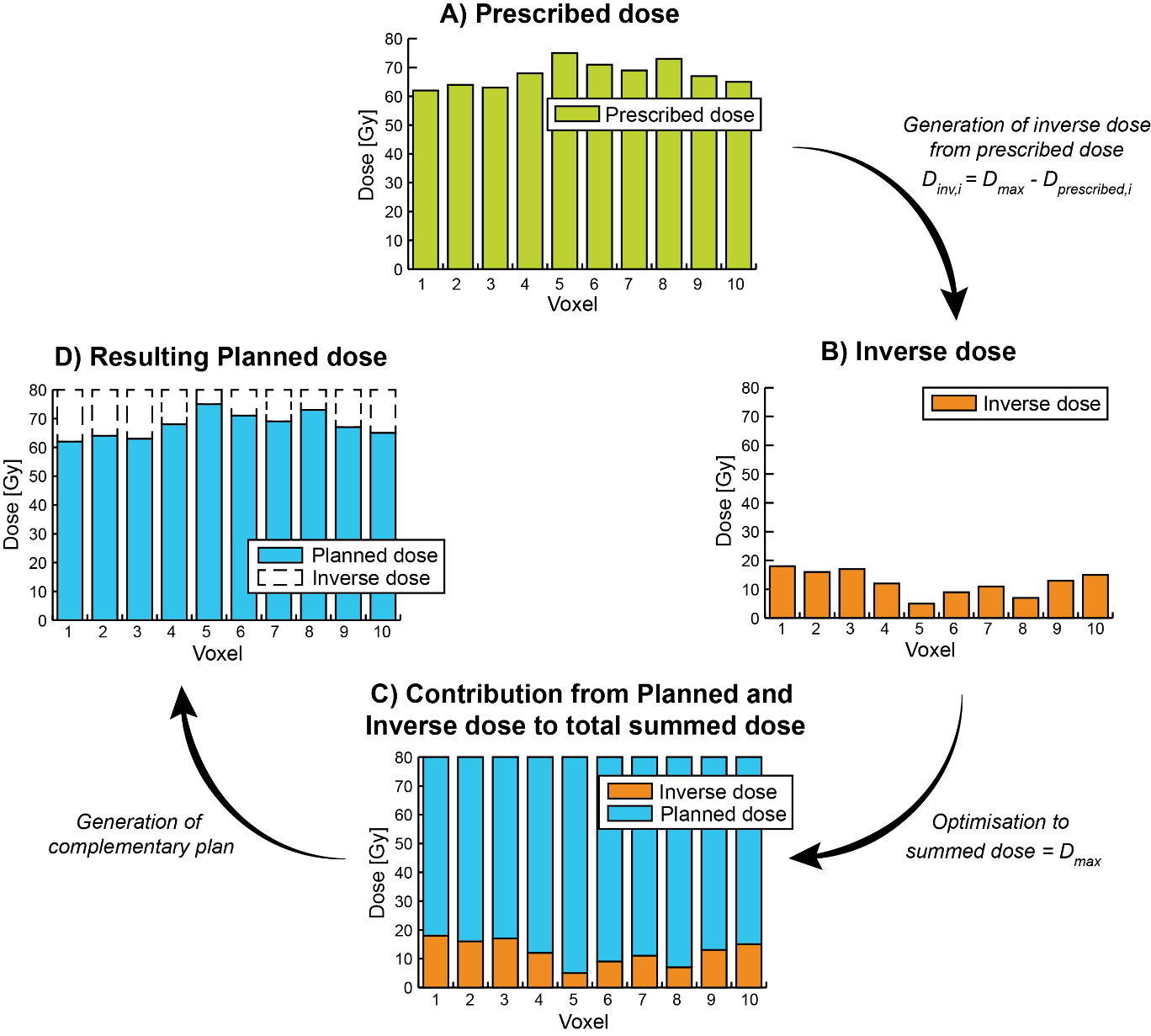


**Figure S1**. *Diagram of inverse dose optimization process*. The inverse dose optimisation process involves three steps. First, the prescribed dose at each voxel, D_prescribed,i_, within the planned target volume is converted into an inverse dose prescription, D_inv,i_ (**A-B**). Second, the inverse dose map is used as a mock plan to optimise the planned dose to an overall maximum dose, D_max_, in dose summation mode (**B-C**). Third, the complementary plan generated from the optimisation process is taken as resulting planned dose (**C-D**). The resulting planned dose should have values close to the values of the prescribed dose.

1. ADC, apparent diffusion coefficient; DSC, dynamic susceptibility contrast enhanced MRI; DWI, diffusion-weighted imaging; FLAIR, fluid-attenuated inversion recovery imaging; FOV, field of view; T1CE, T_1_-weighted contrast enhanced imaging; TE, echo time; TI, inversion time; TR, repetition time, α, flip angle. [↑](#footnote-ref-1)
